# Risk factors and clinical prediction models for low-level viremia in people living with HIV receiving antiretroviral therapy: an 11-year retrospective study

**DOI:** 10.3389/fmicb.2024.1451201

**Published:** 2024-11-01

**Authors:** Wenhui Zhang, Jinchuan Shi, Ying Wang, Er Li, Dingyan Yan, Zhongdong Zhang, Mingli Zhu, Jianhua Yu, Yi Wang

**Affiliations:** ^1^Department of Infection, Hangzhou Xixi Hospital, Zhejiang Chinese Medical University, Hangzhou, China; ^2^Department of Nursing, Hangzhou Xixi Hospital, Zhejiang Chinese Medical University, Hangzhou, China; ^3^Medical Laboratory, Hangzhou Xixi Hospital, Zhejiang Chinese Medical University, Hangzhou, China; ^4^Clinical Research Laboratory, Hangzhou Xixi Hospital, Zhejiang University of Traditional Chinese Medicine, Hangzhou, China

**Keywords:** prediction model, nomogram, viral load, low-level viremia, people living with HIV

## Abstract

**Objective:**

This study explores the risk factors for low-level viremia (LLV) occurrence after ART and develops a risk prediction model.

**Method:**

Clinical data and laboratory indicators of people living with HIV (PLWH) at Hangzhou Xixi Hospital from 5 April 2011 to 29 December 2022 were collected. LASSO Cox regression and multivariate Cox regression analysis were performed to identify laboratory indicators and establish a nomogram for predicting LLV occurrence. The nomogram’s discrimination and calibration were assessed via ROC curve and calibration plots. The concordance index (C-index) and decision curve analysis (DCA) were used to evaluate its effectiveness.

**Result:**

Predictive factors, namely, age, ART delay time, white blood cell (WBC) count, baseline CD4^+^ T-cell count (baseline CD4), baseline viral load (baseline VL), and total bilirubin (TBIL), were incorporated into the nomogram to develop a risk prediction model. The optimal model (which includes 6 variables) had an AUC for LLV after 1-year, 3-year, and 5-year of listing of 0.68 (95% CI, 0.61–0.69), 0.69 (95% CI, 0.65–0.70), and 0.70 (95% CI, 0.66–0.71), respectively. The calibration curve showed high consistency between predicted and actual observations. The C-index and DCA indicated superior prediction performance of the nomogram. There was a significant difference in CD4 levels between LLV and non-LLV groups during the follow-up time. The dynamic SCR, ALT, TG and BG levels and occurrence of complications differed significantly between the high- and low-risk groups.

**Conclusion:**

A simple-to-use nomogram containing 6 routinely detected variables was developed for predicting LLV occurrence in PLWH after ART.

## Introduction

Acquired immunodeficiency syndrome (AIDS) caused by the human immunodeficiency virus (HIV) has long represented a major public health threat (Mallewa et al., 2018). Antiretroviral therapy (ART) effectively inhibits HIV replication leading to virological suppression in most people living with HIV (PLWH) (Reeves et al., 2018). However, in approximately 20–30% of PLWH present with a viral load (VL) of between 50 and 999 copies/mL after 6 months of ART, which, according the World Health Organization (WHO), is defined as low-level viremia (LLV) (Lan et al., 2023; Liu et al., 2024).

Importantly, LLV has been associated with an increased risk of virological failure (Elvstam et al., 2023; Hermans et al., 2018), altered immune status (Ruggiero et al., 2018), emergence of mutations associated with drug resistance (Taramasso et al., 2020; Villalobos et al., 2020), as well as serious AIDS- and non-AIDS-related morbidity and mortality (Elvstam et al., 2021; Li et al., 2021; Galli et al., 2020). Therefore, early diagnosis of LLV is critical for the effective long-term management of PLWH.

Causes and predictors of LLV, as well as its clinical significance are still controversial. Several HIV cohort studies have found that the lower baseline CD4^+^ T cell counts and higher baseline VL are associated with LLV risk (Lombardi et al., 2024; Bai et al., 2022). In addition, Zhang et al. revealed that other risk factors associated with included long-term ART, Manchu (a member of an Indigenous people of Manchuria), and subtype B′ infection (Zhang et al., 2020). Another previous study has shown that in addition to these factors, gender, therapy history, co-receptor tropism, and VL at ART initiation could be valuable predictive markers to identify patients at risk for long-term persistent LLV (Vancoillie et al., 2014). Although numerous predictors related to the occurrence of LLV have been reported, it is imperative to identify novel sensitive markers of LLV.

The progression of viremia in PLWH is complex and involves multiple factors; therefore, the use of a single factor to accurately predict the outcome of LLV is not feasible. Nomogram, as a convenient and useful models for clinicians, integrates multiple key factors, and each relevant parameter is scored based on its value (Chen et al., 2022). In the context of AIDS, the nomograms are frequently applied to assess the risk of HIV infection and the incidence of AIDS and non-AIDS diseases (Yang et al., 2022). With the development of ART in recent years, some nomograms have also been established to predict the mortality and outcome of immune reconstitution of PLWH after ART (Jiang et al., 2022; Wang et al., 2024). Currently, little is known about the utility of nomograms in predicting LLV among PLWH after ART.

In this study, we identified some determinants of LLV among PLWH and developed a simple-to-use nomogram to predict the outcome of LLV after ART in these patients.

## Methods

### Sample size calculation

According previous study, the incidence of LLV was 9% (Elvstam et al., 2021), the sensitivity was 0.9 (Negida et al., 2019), the allowable error L was set at 10%, the test level *α* = 0.05 (bilateral). The sample size of the diagnostic test was calculated according to the sample size formula.


$$\begin{array}{l} N=Z_{\alpha /2}^{2}\times \frac{S_{N}\left(1-S_{N}\right)}{L^{2}\times P}=1.96\times 1.96\times 0.9\left(1-0.9\right)/\\ \;\left(0.1^{2}\times 9\%\right)=384. \end{array}$$

Considering the shedding rate of about 20%, the total sample size required was 461 cases, at least.

### Study population and design

**Figure 1:**
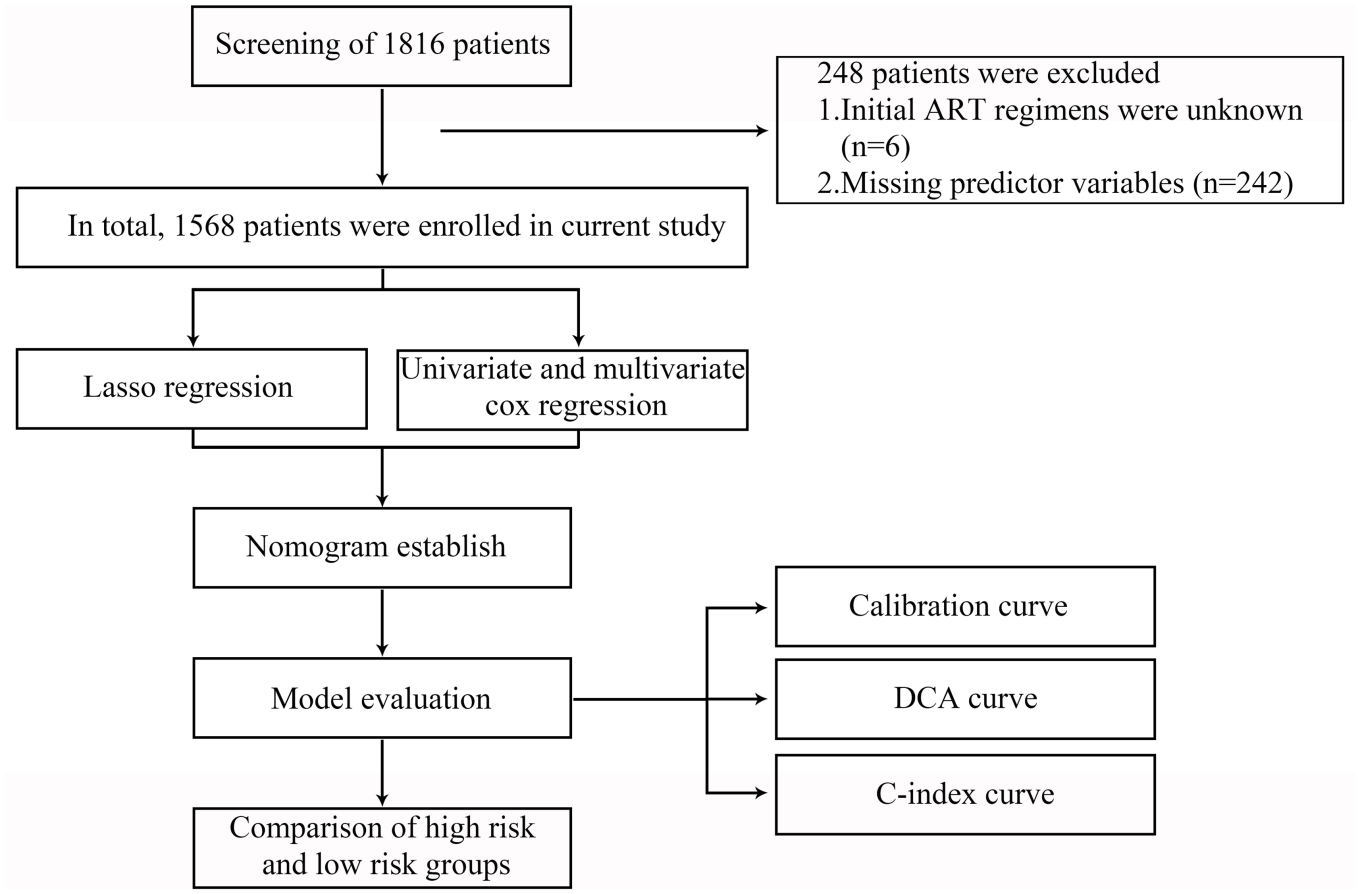
Flowchart of all participants in the study. LASSO, the least absolute shrinkage and selection operator; ART, antiretroviral therapy; DCA, decision curve analysis; C-index, concordance index.

In the initial study, a total of 1816 PLWH were recruited. All enrolled participants received standardized ART treatment between 5 April 2011 and 29 December 2022, at Hangzhou Xixi Hospital, the largest AIDS designated hospital in Zhejiang Province, China. The inclusion criteria were as follows: (1) age > 18 years; (2) have received ART for at least 6 months; (3) the plasma VL was lower than 1,000 copies/mL; (4) followed over the period 2011–2022; (5) with at least two consecutive viremia values/per year, in the period analyzed, carried out at least 1 month apart. The exclusion criteria were as follows: (1) initial ART regimens were unknown; (2) predictor variables were incomplete. The flowchart is shown in Figure 1.

### Outcome definitions

In our study, the outcome of interest was LLV, which was defined as the occurrence of at least two consecutive VL measurements of VL ≥ 50 to ≤999 copies/mL after 6 months of ART.

### Data collection

Data for the following demographic and clinical variables were collected at baseline via a questionnaire: age (pre-ART), sex, marital status, alcohol consumption status, smoking status, body mass index (BMI), monthly salary, education level, route of transmission, hepatitis B infection status, hepatitis C infection status, complications (high blood pressure, hyperglycemia and others), ART initial regimens, use of cotrimoxazole prophylaxis, ART delay time (months, time from HIV diagnosis to ART initiation) (Bogdanić et al., 2021), and time initiated (years). The data of comorbidities were collected at the last patients evaluation. The laboratory parameters included white blood cell (WBC), baseline CD4^+^ T-cell counts, baseline VL, hemoglobin (HB), total bilirubin (TBIL), platelet (PLT), creatinine (CR), aspartate aminotransferase (AST), alanine aminotransferase (ALT), AST/ALT ratio, total cholesterol (TC), total triglyceride (TG), and blood glucose (BG) required to be longitudinally collected (from baseline to the last patients evaluation).

### Predictor selection

The least absolute shrinkage and selection operator (LASSO) Cox method was applied for data dimension reduction and the optimal predictors were selected for the nomogram (Wang et al., 2024). The “glmnet” program package was utilized in the R software, and the “family = Cox” model parameter was selected (Li et al., 2022).

### Construction of nomogram

The predictive model was visualized and presented as a nomogram. The nomogram was drawn based on the results of the multivariate Cox regression analysis which used the “rms” package of R software. To calculate the score for each variable at each level, a scoring standard was developed based on the standard regression coefficients of all variables (Wang et al., 2024). Then, the scores of these factors were used to calculate a total score to reflect the probability of LLV in each patient.

### Statistical analyses

All statistical analysis was performed using the R statistical software version 4.1.3 (R Foundation for Statistical Computing, Vienna, Austria) and IBM SPSS Statistic version 25.0 (IBM, Armonk, NY, USA). Continuous variables are presented as mean and standard deviation (SD). Categorical variables are presented as frequency and percentage, and the percentages were compared using Pearson’s chi-square tests (Zhang et al., 2023). All line graphs were generated using GraphPad Prism version 8.0.2 (263) (GraphPad Software, San Diego, CA, USA).

A multivariable Cox regression model was applied to determine covariate association with LLV. A hazard ratio (HR) of greater or less than 1 was interpreted as an increased or decreased association with LLV, respectively. We used the “Survival” and “Survminer” packages in R to analyze the LLV data from PLWH. The predictive accuracy of the risk model was assessed using the receiver operating characteristic (ROC) curve. The area under ROC curve (AUC) was plotted with the MedCalc software package version 18.2.1 (MedCalc Software, Ostend, Belgium) using sensitivity and specificity values (Kats et al., 2021). The calibration plot was implied by a 45° diagonal line with 1,000 bootstrap samples (Yu et al., 2020; Yuan et al., 2021). The concordance index (C-index) (Wang et al., 2024) and decision curve analysis (DCA) (Vickers et al., 2019) were plotted to evaluate the clinical usefulness and applicability of net benefits of the model with the best predictive value. Kaplan–Meier curves were drawn for PLWH with or without LLV and compared using log-rank tests. A generalized linear mixed model (GLMM) was used to compare the differences in clinical indexes between two groups of PLWH under different follow-up times. All reported levels of statistical significance were two-sided, and *p*-values <0.05 were considered to indicate statistical significance (Wang et al., 2023).

## Results

### Study populations

Based on the inclusion and exclusion criteria, 1,568 PLWH were included in the retrospective analysis (Figure 1). The 248 excluded PLWH comprised 6 patients with unknown initial ART regimens and 242 patients with incomplete clinical data. The mean age of the 1,568 participants was 32.54 years (SD: 11.2); 94.6% were male, while 5.4% were female; and the mean BMI was 22.61 (SD: 3.11). In our study, 11.1% (174/1568) of PLWH had LLV after long-term ART (Table 1). Further details are provided in Table 1.

**Table 1:** Baseline characteristics of all participants (*n* = 1,568).

Characteristics	Values
Sex (%)	
Male	1,484 (94.6)
Female	84 (5.4)
Age (years)	32.54 ± 11.2
Marital status (%)	
Married	1,143 (72.9)
Unmarried	276 (17.6)
Divorced or widowed	149 (9.5)
Education (%)	
Junior and below	203 (12.9)
Senior	226 (14.4)
Junior college	405 (25.8)
Graduate and above	734 (46.8)
Monthly salary (%)	
<5,000 RMB	386 (24.6)
5,000 ~ 10,000 RMB	613 (39.1)
>10,000 RMB	569 (36.3)
Smoking (%)	
Yes	464 (29.6)
No	1,104 (70.4)
Drinking (%)	
Yes	539 (34.4)
No	1,029 (65.6)
HBP (%)	
Yes	88 (5.6)
No	1,480 (94.4)
Complications (%)	
Yes	150 (9.6)
No	1,418 (90.4)
ART regimen (%)	
NNRTIs+NRTIs	1,273 (81.2)
INSTIs+NRTIs	282 (18)
PIs + NRTIs	13 (0.8)
Cotrimoxazole prophylaxis (%)	
Yes	232 (14.8)
No	1,336 (85.2)
Route of transmission (%)	
Homosexual	1,223 (78)
Heterosexual	327 (20.9)
Other (mother-to-child transmission and others)	18 (1.1)
ART delay time (months)	6.06 ± 19.18
Baseline CD4 (cells/μL)	323.3 ± 182.64
Baseline VL (%)	
< 100,000 copies/mL	1,005 (64.1)
100,000 ~ 500,000 copies/mL	337 (21.5)
> 500,000 copies/mL	226 (14.4)
Low-level viremia	
Yes	174 (11.1)
No	1,394 (88.9)
HB (g/L)	146.79 ± 15.99
TBIL (μmol/L)	12.43 ± 5.7
BMI	22.61 ± 3.11
PLT (10^9^/L)	213.13 ± 61.91
WBC (10^9^/L)	5.75 ± 1.82

HBP, high blood pressure; ART, antiretroviral therapy; NNRTIs, non-nucleoside reverse transcriptase inhibitors; NRTIs, nucleoside reverse transcriptase inhibitors; INSTIs, integrase inhibitors; PIs, protease inhibitors; CD4, CD4^+^ T cells; VL, viral load; HB, hemoglobin; TBIL, total bilirubin; BMI, body mass index; PLT, platelet; WBC, white blood cells.

### Selection of risk predictors

**Figure 2:**
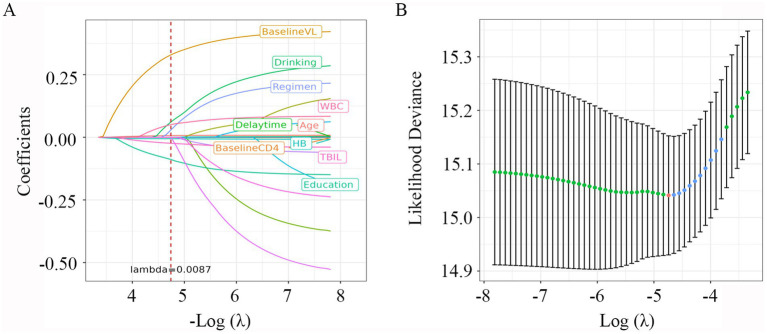
LASSO Cox regression plot. (A) Plot of partial likelihood deviance; (B) plot of LASSO coefficient profiles. Each color curve represents the LASSO coefficient profile of a feature against the Log (*λ*) sequence. The values above the figure represent the numbers of variables included in the model, with the corresponding λ shown on the x-axis. λ, lambda.

Variables with *p* less than 0.1 in the Lasso regression analysis were included in the univariate and multivariable Cox regression. As shown in Figure 2, with the increase of *λ*, the variable coefficients continued to decrease. The optimal harmonic parameter *λ* was selected through cross-validation. In this case, lambda.min was selected at 0.0087, as shown in Figure 2. Finally, seven optimal parameter values were included into the further analysis.

### Establishment of risk score model

We first calculated the cut-off values for six continuous variables that were significantly associated with the outcome of LLV. The optimal cut-off value for age was >30 years, with a sensitivity and specificity of 58.62 and 57.96%, respectively. The baseline CD4 had 61.49% sensitivity and 56.1% specificity at an optimal cut-off value of ≤290 cells/μL. The optimal cut-off value of ART delay time was >1 month, with a sensitivity and specificity of 44.25 and 71.45%, respectively. The HB had 49.43% sensitivity and 67.86% specificity at an optimal cut-off value of ≤143 g/L (Supplementary Table S1). The optimal cut-off value of TBIL was ≤9.8 μmol/L, with a sensitivity and specificity of 54.02 and 65.93%, respectively. The WBC count had 28.74% sensitivity and 77.83% specificity at an optimal cut-off value of >6.86 cells/μL (Supplementary Table S1).

**Figure 3:**
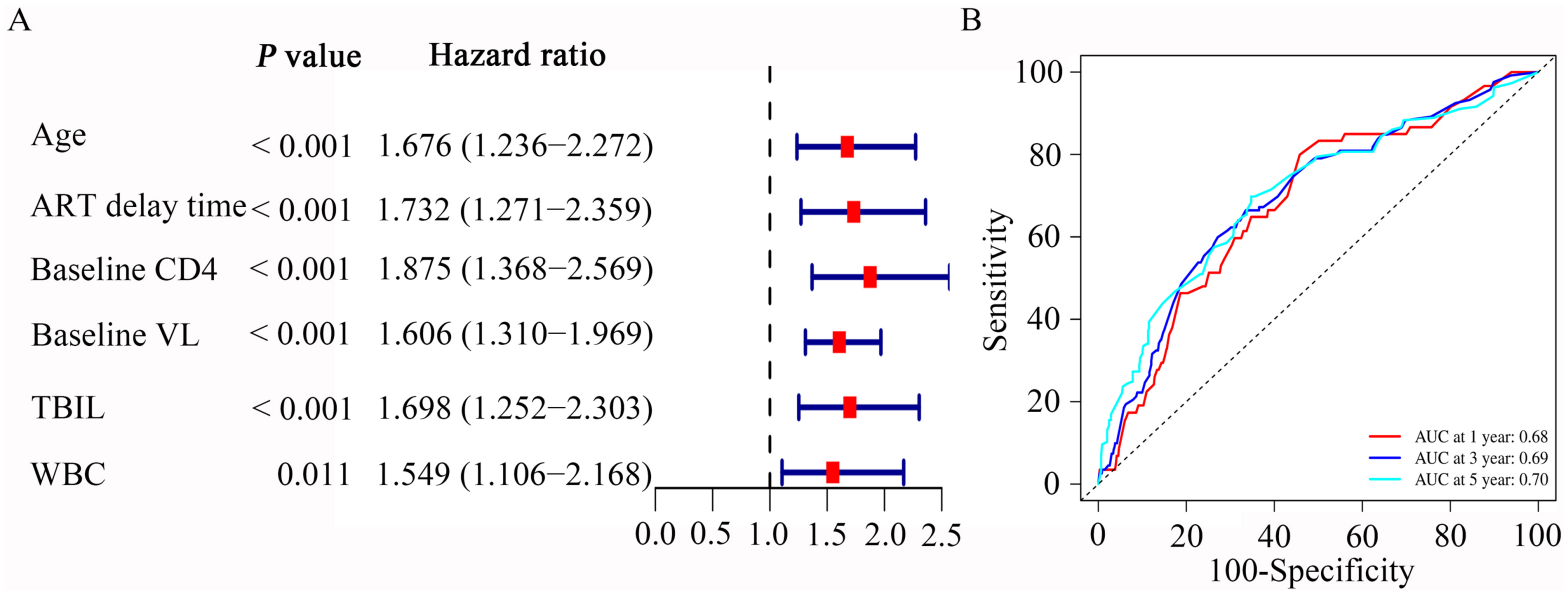
Multivariate Cox regression and ROC curves analysis was used to evaluate the risk factors associated with LLV. (A) Forrest plot of multivariate Cox regression analysis. (B) Time-independent ROC curves of the nomogram for 1-, 3- and 5-year overall survival prediction in the PLWH cohort.

We then used the univariate and multivariable Cox regression analysis to screen out six variables, including age, baseline CD4, ART delay time, baseline VL, TBIL, and WBC count (all *p* < 0.05, Table 2). As in Figure 3A, the forest maps showed that the age (HR = 1.676; 95% CI: 1.236–2.272; *p* < 0.001), ART delay time (HR = 1.732; 95% CI: 1.271–2.359; *p* < 0.001), baseline CD4 (HR = 1.875; 95% CI: 1.368–2.569; *p* < 0.001), baseline VL (HR = 1.606; 95% CI: 1.310–1.969; *p* < 0.001), TBIL (HR = 1.698; 95% CI: 1.252–2.303; *p* < 0.001) and WBC (HR = 1.549; 95% CI: 1.106–2.168; *p* = 0.011), which were significantly associated with LLV. Another study showed that Normal liver function group and Abnormal liver function group among patients with HIV-1 mono-infection receiving ART.

**Table 2:** Univariate and multivariate cox regression analysis for factors associated with the outcome of LLV for PLWH.

Variable	Univariate analysis		Multivariate analysis	
	HR (95CI%)	*p* value	HR (95CI%)	*p* value
Age (> 30 years)	1.83 (1.353–2.475)	< 0.0001	1.614 (1.187–2.195)	0.002
Baseline CD4 (≤ 290 cells/μL)	1.952 (1.438–2.650)	< 0.0001	1.780 (1.291–2.254)	< 0.0001
ART delay time (> 1 month)	1.464 (1.082–1.980)	0.013	1.742 (1.278–2.373)	< 0.0001
Baseline VL	1.563 (1.281–1.907)	< 0.0001	1.585 (1.293–1.945)	< 0.0001
TBIL (≤ 9.8 μmol/L)	1.940 (1.438–2.616)	< 0.0001	1.585 (1.157–2.171)	0.004
WBC (> 6.86 cells/μL)	1.395 (1.005–1.938)	0.047	1.547 (1.105–2.167)	0.008
HB (≤ 143 g/L)	1.851 (1.374–2.493)	< 0.0001	1.333 (0.968–1.836)	0.078

HR, hazard ratio; CI, confidence interval; CD4, CD4^+^ T cells; ART, antiretroviral therapy; CD4, CD4^+^ T cells; VL, viral load; TBIL, total bilirubin; WBC, white blood cells; HB, hemoglobin.

We then performed ROC analysis to assess the ability of different models consisting of 6 independent variables to predict the outcome of LLV among PLWH. Table 3 compared the AUC values of five models for 1-year, 3-year and 5-year occurrence of LLV in PLWH cohort. The combined model 1 (includes six variables) had an AUC for LLV after 1-year, 3-year and 5-year of listing of 0.68 (95% CI, 0.61–0.69), 0.69 (95% CI, 0.65–0.70) and 0.70 (95% CI, 0.66–0.71), respectively (Table 3). The combined model 2 (includes five variables, except for age) had an AUC for LLV after 1-year, 3-year and 5-year of listing of 0.69 (95% CI, 0.64–0.70), 0.69 (95% CI, 0.65–0.70) and 0.69 (95% CI, 0.66–0.71), respectively (Table 3). The combined model 3 (includes five variables, except for WBC) had an AUC for LLV after 1-year, 3-year and 5-year of listing of 0.67 (95% CI, 0.60–0.68), 0.68 (95% CI, 0.63–0.69) and 0.68 (95% CI, 0.65–0.70), respectively (Table 3). The combined model 4 (includes five variables, except for TBIL) had an AUC for LLV after 1-year, 3-year and 5-year of listing of 0.64 (95% CI, 0.58–0.65), 0.67 (95% CI, 0.63–0.68) and 0.70 (95% CI, 0.66–0.71), respectively (Table 3). The combined model 5 (includes five variables, except for delay time) had an AUC for LLV after 1-year, 3-year and 5-year of listing of 0.66 (95% CI, 0.59–0.67), 0.67 (95% CI, 0.63–0.69) and 0.69 (95% CI, 0.66–0.71), respectively (Table 3). Given the model with fewer variables was more susceptible to underfitting and the optimal prediction model should include statistically significant variables, the combined model 1 containing age, baseline CD4, ART delay time, baseline VL, TBIL, and WBC was used to evaluate the occurrence of LLV. Plots of time-dependent for 1-year, 3-year and 5-year AUCs are shown in Figure 3B.

**Table 3:** Predictive values of different models for outcome of LLV after 1, 3, 5 years.

Model	Variable number	1-year AUC (95% CI)	3-year AUC (95% CI)	5-year AUC (95% CI)
Model1	6	0.68 (0.61–0.69)	0.69 (0.65–0.70)	0.70 (0.66–0.71)
Model2	5 (no Age)	0.69 (0.64–0.70)	0.69 (0.65–0.70)	0.69 (0.66–0.71)
Model3	5 (no WBC)	0.67 (0.60–0.68)	0.68 (0.63–0.69)	0.68 (0.65–0.70)
Model4	5 (no TBIL)	0.64 (0.58–0.65)	0.67 (0.63–0.68)	0.70 (0.66–0.71)
Model5	5 (no Delay time)	0.66 (0.59–0.67)	0.67 (0.63–0.69)	0.69 (0.66–0.71)

AUC, area under the curve; CI, confidence interval; WBC, white blood cells; TBIL, total bilirubin.

**Figure 4:**
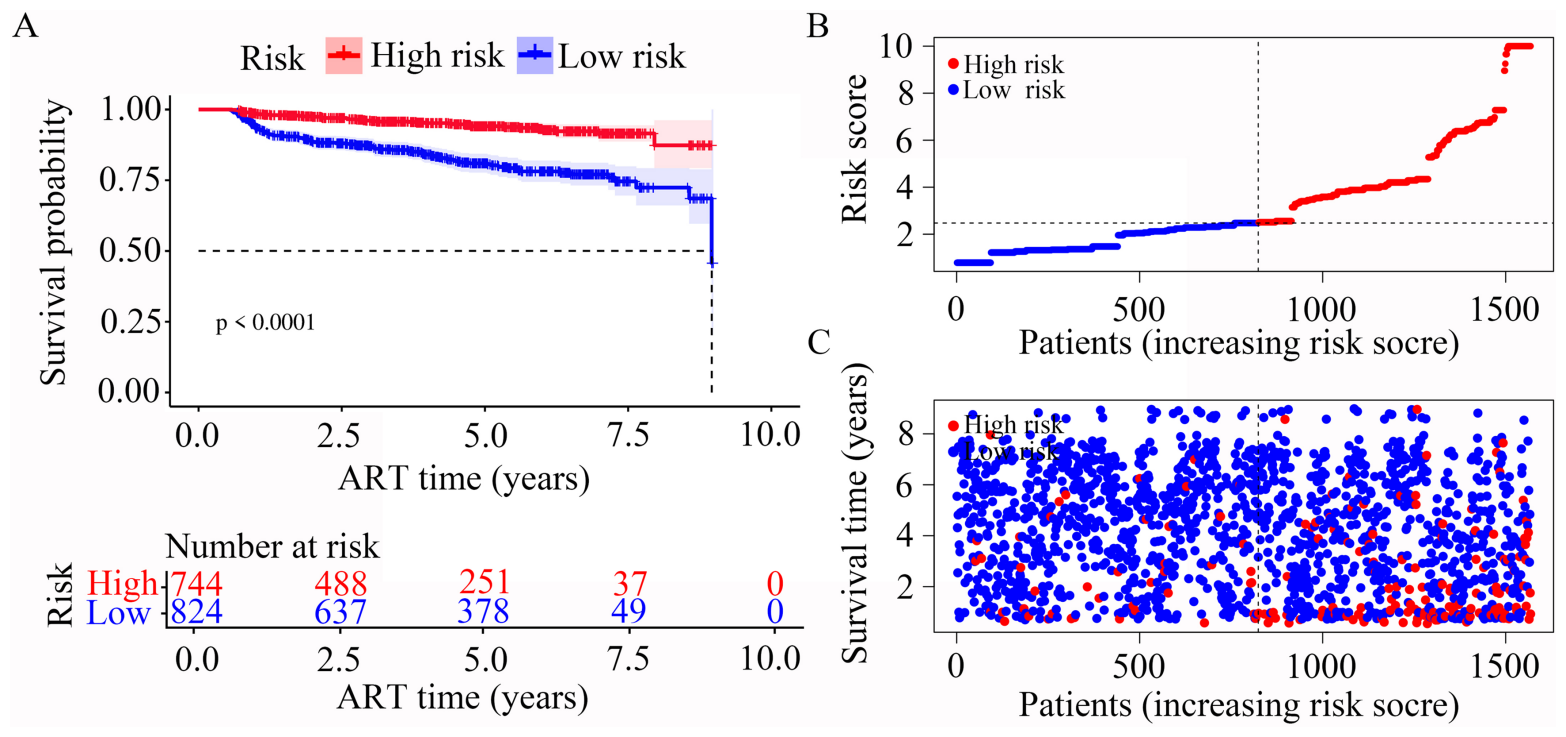
Construction of the risk score model with respect to PLWH with LLV. **(A)** Kaplan–Meier curves of the overall survival of the two LLV risk groups of PLWH with LLV. **(B,C)** Risk score curves and scatter plots of the risk of LLV among the PLWH cohorts.

In our risk model, PLWH were divided into two groups according to risk (high vs. low), and the grouping criterion was the median of the risk score. Subsequent survival analysis revealed that the outcome of LLV differed significantly between the two groups (Figure 4A, *p* < 0.0001). The risk curve shows the relationship between LLV and the risk of PLWH. Figure 4B shows the risk values of PLWH in the two groups. PLWH in the high-risk group had significantly higher incidence of LLV than those in the low-risk group (Figure 4C).

### Performance and evaluation of risk nomogram

**Figure 5:**
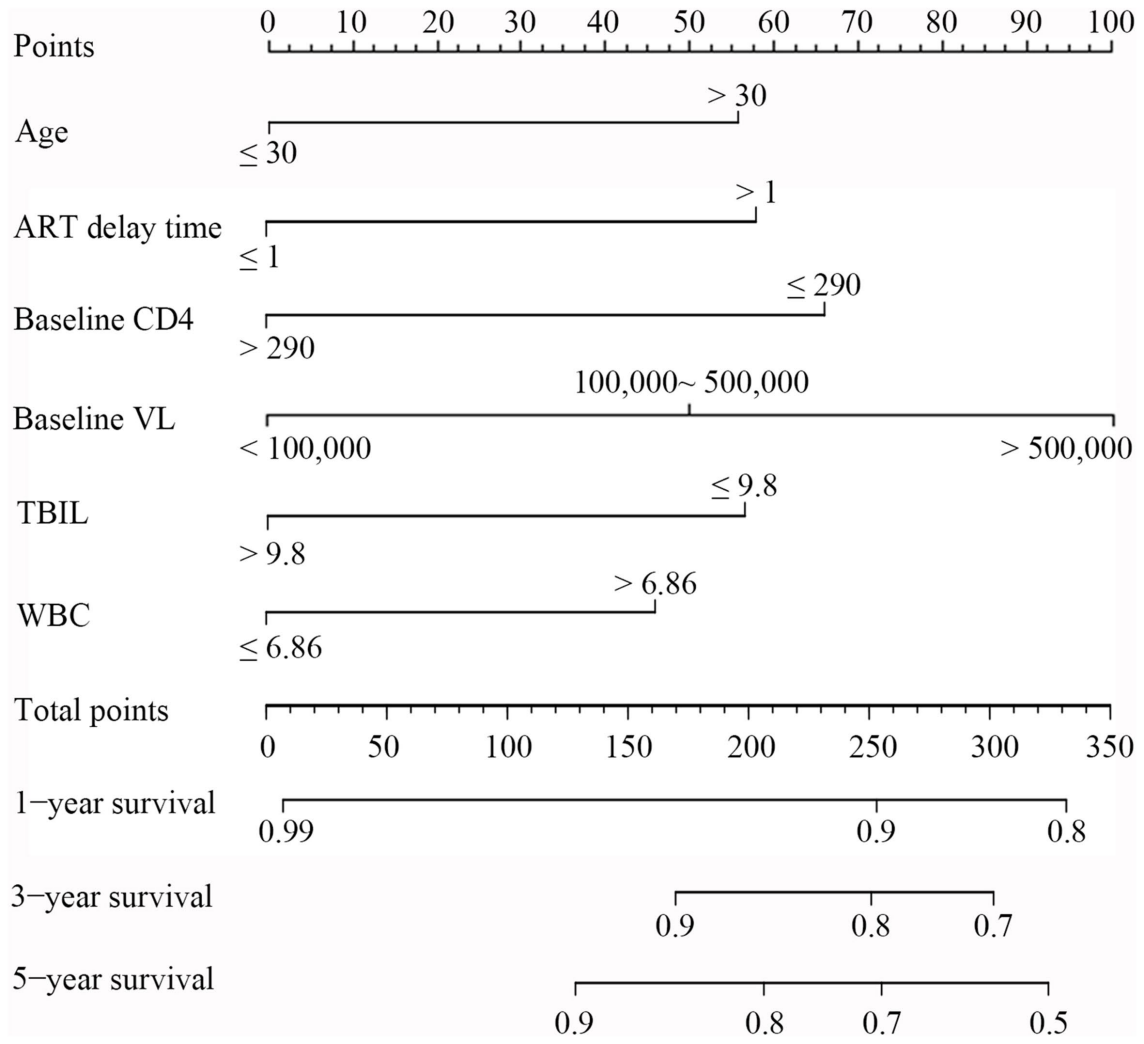
Nomogram for evaluation of LLV in PLWH. To use the nomogram, a line was first drawn from each parameter value to the score axis for the score; then the points for all the parameters were added. Finally, a line from the total score axis was drawn to determine the occurrence of LLV on the lower line of the nomogram.

Based on the above results, 6 variables, including age, baseline CD4, ART delay time, baseline VL, TBIL, and WBC, were incorporated into a risk predictive nomogram for predicting the occurrence of LLV among PLWH. Figure 5 shows the protocol for the use of the risk nomogram. The score of each predictive factor could be calculated, and then the total score was calculated as the sum of scores of 6 predictors. In our model, PLWH with higher score represented the higher risk of LLV after ART.

**Figure 6:**
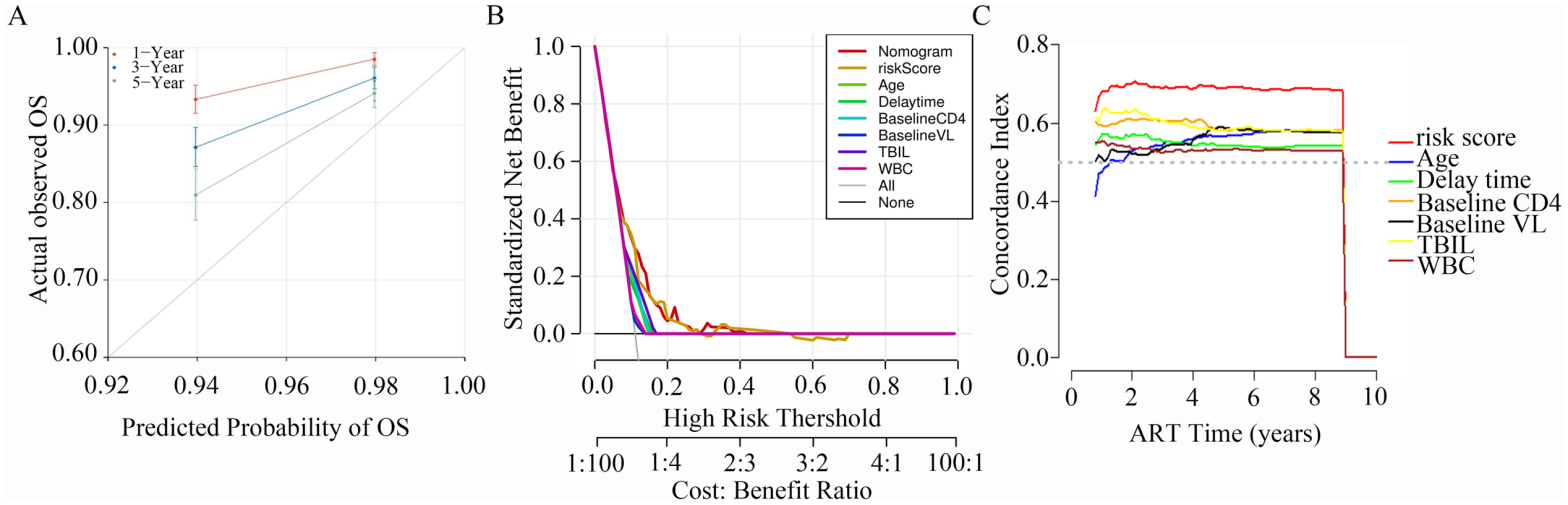
Calibration plots, DCA curves, C-index for the predictive nomogram. (A) Calibration curves for 1-, 3- and 5-year survival depict the calibration of nomogram in terms of the agreement between the predicted probabilities and observed outcomes of PLWH cohort. (B) The results of DCA analysis for the nomogram. (C) Time-dependent C-index of the nomogram model.

Calibration plots were constructed to assess the concordance between predicted and actual outcomes (Yuan et al., 2020). Our calibration curves for each model at 1, 3, and 5 years are shown in Figure 6A. The calibration plots exhibited a high conformance in predicting the occurrence of LLV in the PLWH cohort. In addition, DCA has been widely used to evaluate the efficacy of specific clinical approaches in many studies (Kerr et al., 2016). Our DCA curve revealed that the risk score provided a greater net benefit compared with the clinical model across a wide range (17 to 70%) of risk thresholds (Figure 6B). Across the 1,000 bootstrap resamples, the optimism-corrected C-index for the risk nomogram was close to 0.7 (Figure 6C), which was a more accurate and robust estimate of performance.

### Dynamic changes in CD4, WBC and TBIL levels between the LLV and non-LLV groups following ART

**Figure 7:**
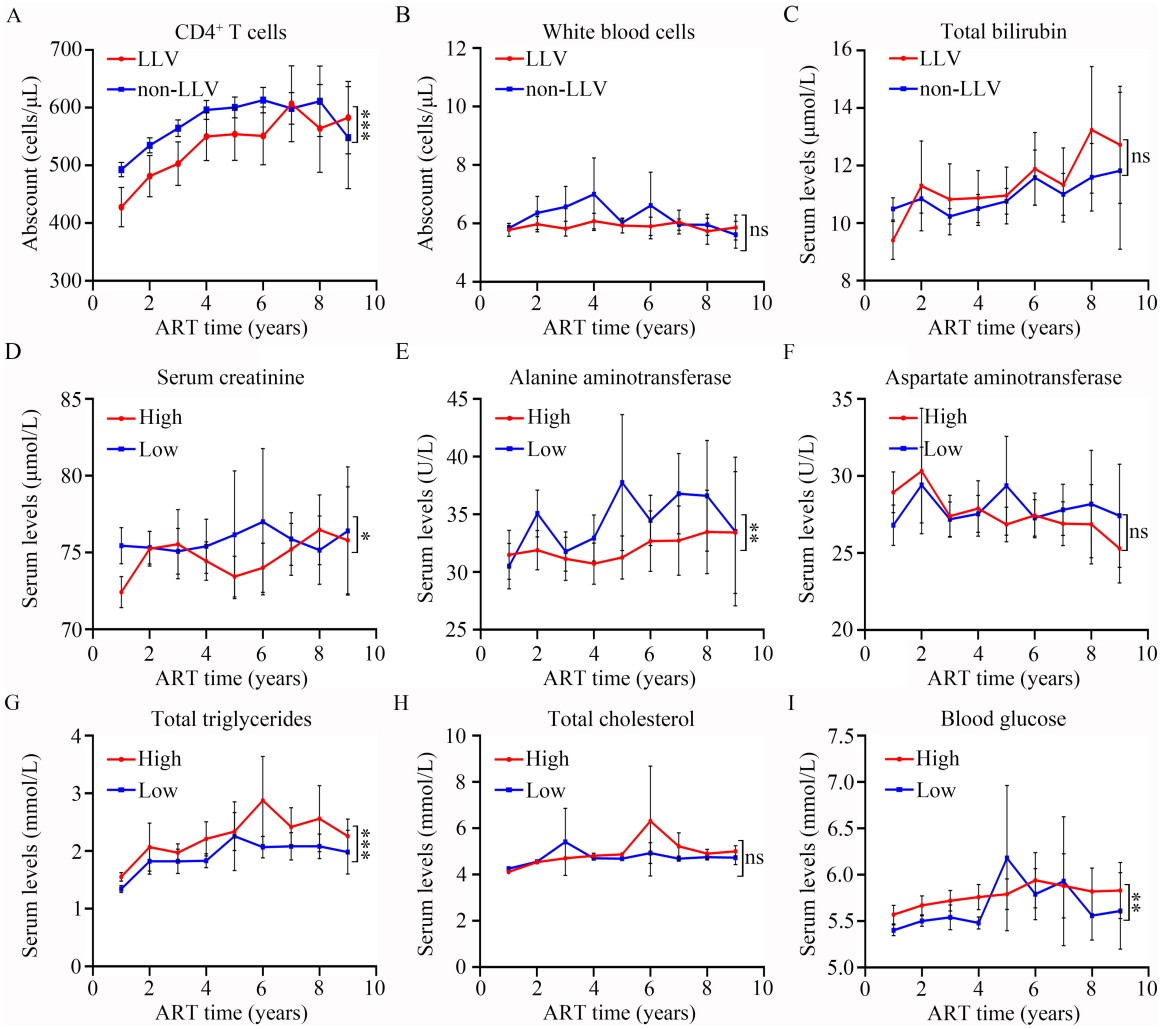
Comparisons of the differences in clinical indexes between different groups after ART. The levels of CD4^+^ T cells (A), white blood cells (B), total bilirubin (C) at different follow-up time points in LLV and non-LLV groups. Serum levels of creatinine (D), alanine aminotransferase (E), aspartate aminotransferase (F), total triglycerides (G), total cholesterol (H), and blood glucose (I) at various different follow-up time points in high-risk and low-risk groups. Wilcoxon matched-pairs signed-rank test with two-tailed *p*-value was used for comparison between groups. ns: *p* > 0.05, *: *p* < 0.05, **: *p* < 0.01, ***: *p* < 0.001.

GLMM analysis showed that there was a significant difference in CD4 levels (*p* < 0.001, Figure 7A) between LLV and non-LLV groups from T1 to T9 after ART. While the difference in the levels of WBC (*p* > 0.05, Figure 7B) and TBIL (*p* > 0.05, Figure 7C) between LLV and non-LLV groups was not obvious during the follow-up time (Supplementary Table S2).

### Dynamic changes in metabolic indexes after ART among high and low risk groups

Based on the risk value, all PLWH were divided into two groups: high risk of LLV, comprising 744 patients; and low risk of LLV, comprising 824 patients. A GLMM analysis was performed to compare the differences in SCR, ALT, AST, TG, TC, and BG at different times between the two groups. As shown in Figure 7 and Supplementary Table S3, the levels of SCR (*p* = 0.01, Figure 7D), and ALT (*p* = 0.004, Figure 7E) in the LLV high-risk group were significantly lower than those in the low-risk group. The levels of TG (Figure 7G). and BG (Figure 7I) among high-risk PLWH were dramatically higher than in the low-risk PLWH (Both *p* < 0.05). However, there was no significant difference in the levels of AST (*p* > 0.05, Figure 7F) and TC (*p* > 0.05, Figure 7H) between the two groups (see Supplementary Table S3).

In addition, we analyzed the differences in the occurrence of various complications between the two groups. As shown in Table 4, there was a significant difference in the occurrence of high TC (*p* = 0.029), high TG (*p* = 0.001), and other complications (including diabetes, coronary heart disease, malignant tumors, and fatty liver) (*p* = 0.002) between the high-risk and low-risk groups. The occurrence of high BG in high-risk group was comparable to those in the low-risk group (Both *p* > 0.05, Table 4).

**Table 4:** Comparisons of the complications between high-risk and low-risk groups.

Variable	High risk (*n* = 744)	Low risk (*n* = 824)	*p* value
HBP, *n* (%)			
No	697 (93.7)	783 (95.0)	0.249
Yes	47 (6.3)	41 (5.0)	
High TC, *n* (%)			
No	539 (72.4)	638 (77.4)	0.029
Yes	200 (26.9)	183 (22.2)	
Unknown	5 (0.7)	3 (0.4)	
High TG, *n* (%)			
No	405 (54.4)	515 (62.5)	0.001
Yes	334 (44.9)	306 (37.1)	
Unknown	5 (0.7)	3 (0.4)	
High BG, *n* (%)			
No	730 (98.1)	812 (98.5)	0.488
Yes	10 (1.4)	8 (1.0)	
Unknown	4 (0.5)	4 (0.5)	
Other complications (including diabetes, coronary heart disease, malignant tumor, fatty liver and others), *n* (%)			
No	690 (92.7)	793 (96.2)	0.002
Yes	54 (7.3)	31 (3.8)	

HBP, high blood pressure; TC, total cholesterol; TG, triglyceride; BG, blood glucose.

## Discussion

In this retrospective study, several predictors associated with LLV in PLWH were identified via the LASSO-Cox and multivariable Cox regression analysis. Among these, age, ART delay time, baseline CD4, baseline VL, TBIL, and WBC count were used to construct a predictive nomogram for the prediction of the outcome of LLV among PLWH after ART. The calibration plots, decision curve, and clinical impact curve demonstrated that the novel model possessed excellent discrimination and calibration performance, and the internal verification indicated the favorable applicability and extrapolation capability of this model.

LLV is a well documented independent risk factor for virologic failure (Li et al., 2021; Joya et al., 2019). Previous studies have shown that higher baseline VL is consistently associated with increased rates of LLV (Elvstam et al., 2021; Chen et al., 2021; Álvarez et al., 2023). For example, Hortensia et al. reported that baseline HIV-1 RNA > 100,000 copies/mL is an independent predictor of higher risk of LLV (Álvarez et al., 2022). In addition, Zhang et al. found that the zenith baseline viral load (VL) above 6 log copies/mL is strongly associated with high-risk LLV (Zhang et al., 2020). Our study is in agreement with these previous studies. Our data showed that the risk scores of LLV in PLWH with baseline VL > 500,000 copies/mL and 100,000 ~ 500,000 copies/mL were significantly higher than those in PLWH with baseline VL < 100,000 copies/mL. These data support that baseline VL is an independent risk factor for LLV.

The present study and previous studies have both confirmed that lower baseline CD4 level is a risk factor associated with LLV (Bai et al., 2022; Lao et al., 2024). In addition, our results found a significant higher risk of LLV in PLWH with baseline CD4 ≤ 290 cells/μL than in those with CD4 > 290 cells/μL. Zhang et al. found that nadir baseline CD4^+^ T cell counts below 200 cells/μL were clearly associated with high risk of LLV (Zhang et al., 2020). These findings highlight that initiation of ART at an early stage among PLWH with high baseline CD4^+^ T-cell counts may avoid the occurrence of LLV and enhance their quality of life.

Compared with PLWH without LLV, those with LLV were more likely to be older at ART initiation (Joya et al., 2019). Our results also showed a significantly higher risk of LLV in PLWH who were > 30 years at ART initiation. However, Wu et al. revealed that the older PLWH (> 50 years) were more likely to have poorer immune responses to HIV treatment than younger PLWH (18–49 years) (Wu et al., 2019). Although this difference could be attributed to the fact that 38.2% of PLWH were above 50 years of age in Wu et al.’s study, while our study population was younger. These findings suggest that older PLWH should be more closely monitored for the occurrence of LLV.

The above three predictors have been reported to be closely linked with LLV, and our data further support the previous findings. In addition, we identify novel predictors of LLV in PLWH. We found that PLWH with ART delay time > 1 month had significantly higher risk of LLV than PLWH with ART delay time ≤ 1 month. These data demonstrate that the longer the delay in initiating ART, the higher the risk of LLV. Previous studies have only suggested that the time from HIV diagnosis to ART initiation is associated with LLV based on univariate analysis, without performing multivariate analysis (Bai et al., 2022). Our data implied the importance of timely diagnosis of HIV infection and initiation of ART at an early stage.

A previous study has shown that serum TBIL levels are higher in ART-naive PLWH than in HIV-positive patients receiving first- and second-line ART (Kolgiri et al., 2018). Our results indicate that there is a significantly higher risk of LLV in PLWH with TBIL ≤9.8 μmol/L group than in those with TBIL >9.8 μmol/L. Therefore, serum TBIL, being an easy-to-measure routine clinical indicator, may serve as a predictor of LLV occurrence.

Previous study showed that the WBC count is lower in PLWH with high VL compared with that in PLWH with low VL (Lin et al., 2019). Our results verified that the risk of LLV in PLWH with WBC counts >6.86 cells/μL was higher than in those with WBC count ≤6.86 cells/μL. These data imply that the WBC count is a risk factor for the occurrence of LLV in PLWH. Of note, considering that WBC detection is simple and widely used in clinical practice, dynamic monitoring of WBC count would be a reliable and convenient strategy for assessment of the risk of LLV occurrence.

In the present study, the above six predictors (age, ART delay time, baseline CD4, baseline VL, TBIL, and WBC count) were combined to construct the predictive models. The present study is the first to report the construction of a nomogram for the prediction of LLV among PLWH after ART. Previous studies have only reported a close relationship between baseline CD4 or baseline VL or age at ART initiation and virologic failure in PLWH after ART. Our results indicate that these factors contribute to the process of LLV. Moreover, our study shows, for the first time, that ART delay time, TBIL, and WBC count are also independent predictors of LLV. Follow-up studies are needed to delve deeper into their roles and mechanisms in LLV. On the other hand, Calibration curves, receiver operating characteristic (ROC) curves, and consistency indexes (C-indexes) were used to estimate the performance of the model (Li et al., 2023; Bai et al., 2018). Decision curve analysis (DCA) curves were used to assess the clinical utility of the model. Our calibration plots (1-, 3-, 5- years), DCA curve and optimism-corrected C-index revealed that this model had excellent performance and clinical utility. The result showed that the nomogram was effective for predicting the occurrence of LLV in the PLWH cohorts. Notably, all predictors in our nomogram can be routinely detected in clinical practice. Thus, detection of these parameters is easy and cost-effective, which makes the dynamic assessment of LLV feasible in PLWH and is also helpful for the optimization of therapies.

The nomogram combining the above 6 risk factors not only showed good performance in terms of clinical prediction, but also enabled the categorization of patients into high- and low-risk groups. Furthermore, differences in the occurrence of high cholesterol, high TG, and other complications (including diabetes) between high- and low-risk groups was observed. Previous studies have shown that persistent viremia is a significant predictor of the development of metabolic syndrome (Squillace et al., 2009). The most common metabolic abnormality among PLWH was hypertriglyceridemia (Ang et al., 2021). These data support that PLWH with high risk of LLV were more likely to develop metabolic syndrome. In other words, PLWH at high risk of LLV need more individualized treatment.

Some limitations of this research should be noted. First, this study was a single-center retrospective analysis, and the nomogram requires further optimization and validation in multi-center PLWH populations. Second, the variables dataset has a missing rate of over 13%, which means that data on vital clinical parameters such as immune cells subpopulations and drug resistance status were not included. It is important incorporate these missing parameters in future studies to enhance the accuracy of the prediction model. Finally, prospective cohort studies should be performed to validate the model in subsequent research.

## Conclusion

Our study identifies patient age, ART delay time, baseline CD4, baseline VL, TBIL, and WBC count as risk factors for the occurrence of LLV among PLWH after ART. In this research, we methodically developed an innovative nomogram, using these 6 clinical risk indicators, to predict the occurrence of LLV risk in PLWH. The constructed nomogram model enables individualized diagnostic analysis and is helpful in developing risk-adapted therapy for PLWH.

## Data Availability

The data analyzed in this study is subject to the following licenses/restrictions: the datasets generated during and/or analyzed during the current study are available from the corresponding author on reasonable request. Requests to access these datasets should be directed to Yi Wang, wangyi_xixi@126.com.
