# Supplementary material for: Risk factors and clinical prediction models for low-level viremia in people living with HIV receiving antiretroviral therapy: an 11-year retrospective study
Source: Front Microbiol. 2024 Nov 1;15:1451201. doi: 10.3389/fmicb.2024.1451201 (PMC11563986; doi:10.3389/fmicb.2024.1451201)
Supplement: Supplementary file 1 [file Data_Sheet_1.pdf]

Table S1. The cutoff values of predictors of outcome of LLV for PLWH.

Variable	Associated criterion	Sensitivity (%)	Specificity (%)	*P* value
Age (years)	> 30	58.62	57.96	0.0002
Baseline CD4 (cells/μL)	≤ 290	61.49	56.1	< 0.0001
ART delay time (months)	> 1	44.25	71.45	0.0048
HB (g/L)	≤ 143	49.43	67.86	< 0.0001
TBIL (μmol/L)	≤ 9.8	54.02	65.93	< 0.0001
WBC (cells/μL)	> 6.86	28.74	77.83	0.6616

Note: CD4: $CD4^+$ T cells; ART: antiretroviral therapy; HB: hemoglobin; TBIL: total bilirubin; WBC: white blood cells.

Table S2. Comparisons of the clinical indexes between LLV and non-LLV groups at different follow-up time after ART.

Variable	β value (95%CI)	*P* value
CD4	51.259 (36.873~65.645)	< 0.001
WBC	-0.008 (-0.164~0.148)	0.921
TBIL	0.282 (-0.154~0.678)	0.217

Note: CI: confidence interval; CD4: $CD4^+$ T cells; WBC: white blood cells; TBIL: total bilirubin.

Table S3. Comparisons of the metabolic indexes between high-risk and low-risk groups at different follow-up time after ART.

Variable	β value (95%CI)	*P* value
SCR	1.056 (0.255~1.857)	0.01
ALT	1.703 (0.557~2.849)	0.004
AST	-0.197 (-0.981~0.587)	0.622
TG	-0.237 (-0.318~ -0.157)	< 0.001
TC	-0.022 (-0.074~0.029)	0.398
BG	-0.197 (-0.259~-0.134)	< 0.001

Note: CI: confidence interval; SCR: serum creatinine; ALT: alanine aminotransferase; AST: aspartate transaminase; TG: triglyceride; TC: total cholesterol; BG: blood glucose.
